# A Multi-Pathogen Retrospective Study in Patients Hospitalized for Acute Gastroenteritis

**DOI:** 10.3390/diseases12090213

**Published:** 2024-09-12

**Authors:** Antonella Zizza, Marcello Guido, Raffaella Sedile, Marzia Benelli, Milva Nuzzo, Pasquale Paladini, Anacleto Romano, Pierfrancesco Grima

**Affiliations:** 1 Institute of Clinical Physiology, National Research Council, 73100 Lecce, Italy; raffaella.sedile@cnr.it; 2 Laboratory of Hygiene, Department of Biological and Environmental Sciences and Technologies, University of Salento, 73100 Lecce, Italy; marcello.guido@unisalento.it; 3 Pediatric Unit, Vito Fazzi Hospital, 73100 Lecce, Italy; marzia.benelli@yahoo.it (M.B.); pediatria.polecce@asl.lecce.it (P.P.); 4 Infectious Diseases Unit, Vito Fazzi Hospital, 73100 Lecce, Italy; nuzzo.milva@tiscali.it (M.N.); malattieinfettive.polecce@asl.lecce.it (A.R.); pierfrancescogrima@yahoo.it (P.G.)

**Keywords:** acute gastroenteritis, hospitalization, vaccination, foodborne disease

## Abstract

Acute gastroenteritis (AGE) is a gastrointestinal tract disease often caused by consuming food or water contaminated by bacteria, viruses, or parasites, that can lead to severe symptoms requiring hospitalization. A retrospective study on patients admitted for AGE between 2021 and 2023 at the Pediatrics and Infectious Diseases Departments of Lecce Hospital was conducted. Demographic characteristics, year and month of admission, length of hospital stay, etiological agents, co-infections, and blood chemistry data of patients were collected. The study included 103 patients ranging in age from 0 to 15 years, with 58.25% being male. A total of 78 bacterial, 35 viral, and 7 parasitic infections were identified. The most commonly detected pathogens were *Escherichia coli* (38.83%), Norovirus (28.16%), *Campylobacter jejuni* (22.33%), and *Salmonella typhi/paratyphi* (10.68%). Only a few cases of *Cryptosporidium* (5.83%) were identified. Additionally, 17 co-infections (16.50%) were detected. Viral infections are the primary cause of hospitalization for AGE in children <5 years, while bacterial infections are more common among older patients. The significantly higher number of children <5 years old with elevated creatinine compared to children ≥5 years suggested that young children are more susceptible to dehydration than older children. Few cases of AGE were attributed to pathogens for which a vaccine has already been licensed. AGE is a serious health concern that could be effectively prevented by implementing food-based and community-level sanitation systems, as well as by increasing vaccination coverage of available vaccines and developing new effective and safe vaccines.

## 1. Introduction

Acute gastroenteritis (AGE) is an inflammation of the gastrointestinal tract, typically caused by consuming contaminated foods or beverages with bacteria, viruses, or parasites, characterized usually by mild symptomatology lasting a couple of days, including abdominal pain, nausea, vomiting, and diarrhea. Although the mortality rate from AGE has decreased in developed countries, morbidity continues to pose challenges, leading to numerous hospitalizations, outpatient evaluations, and related costs [1,2,3].

The population at risk for severe AGE includes children under 5 years of age, the elderly (over 65 years old), and immunocompromised individuals (such as those affected by HIV or with a history of organ transplant), who are more vulnerable to severe dehydration [1]. The Global Burden of Disease (GBD), which provides data from 195 countries, reports that diarrhea is the eighth leading cause of death among individuals of all ages, with 4.4 billion cases and 1,655,944 deaths (95% uncertainty interval [UI]: 1,244,073–2,366,552) and the fifth leading cause of death among children under 5 years of age with 446,000 deaths (95% UI: 390,894–504,613) [4].

Enteric infections are usually resolved without medical intervention and therefore not notified [5]. Only in cases that result in hospitalization is the cause of the disease further investigated. Globally, viruses are the primary cause of gastroenteritis, accounting for 50–70% of cases, followed by bacteria for 15–20%, and parasites responsible for 10–15% of cases [6].

Over 20 different viruses have been identified as etiologic agents of AGE [7,8]. The most widespread viruses are Rotaviruses, Noroviruses, Adenoviruses, and Astroviruses, with prevalence varying depending on age groups and epidemic season, as well as geographical, environmental, and socioeconomic factors [9].

Rotaviruses and noroviruses are responsible for about 35–40% of hospital admissions for diarrhea in infants and young children, causing 200,000 deaths worldwide, with higher prevalence in developing countries, where access to rehydration care is limited than in high-income countries [10,11]. Noroviruses are the main cause of AGE in people across all ages responsible for 18% of all clinical cases [12]. In adults, they are followed by rotavirus, adenovirus, and astrovirus [13], while among younger patients, rotavirus is the most prevalent followed by norovirus and adenovirus. However, in some regions, the introduction of vaccines has reduced AGE morbidity due to certain etiological agents [14,15,16,17,18].

In countries where children are effectively immunized against rotavirus, norovirus is now the leading pathogen associated with medically attended cases of AGE [12,19,20].

Among the bacteria responsible for AGE, the most commonly encountered are *Salmonella*, *Shigella*, enterotoxigenic *Escherichia coli* (ETEC), and *Campylobacter* spp., while *Cryptosporidium* spp. are the most frequent parasitic pathogens [21].

*Campylobacter* is the most frequently reported bacterial pathogen in the EU, with over 137,000 cases in 2022 and a 23.5% hospitalization rate [22].

The use of antimicrobial drugs to reduce the spread of these infections has the dual effect of increasing the risk of antimicrobial resistance. The development of resistance, coupled with the difficulty in accessing these medicines in some countries, makes treating and controlling the infection more difficult. Vaccination is widely acknowledged as the most effective and practical means of preventing enteric infections and reducing antimicrobial resistance. Developing vaccines to control AGE is a global public health priority, as highlighted by the WHO [23,24,25].

At present, only vaccines against typhoidal *Salmonella*, rotavirus, and *Vibrio cholerae* are available, but there are no authorized vaccines for parasites or many other enteric viral and bacterial pathogens such as calicivirus, diarrheal *E. coli*, *Shigella*, *Campylobacter*, and non-typhoid *Salmonella*. However, progress has been made in developing vaccines for some of these pathogens, with some in an advanced stage of human trials, so much so that effective and practical vaccines could soon be available to prevent a significant portion of diarrheal disease [26].

Recent advancements in the vaccine pipeline for *Shigella* and ETEC, have prompted the WHO to reaffirm these pathogens as priority vaccine targets [27].

The incidence of AGE displays a clear seasonality, with peaks occurring during autumn and winter for viral infections and during the summer for acute bacterial gastroenteritis [6,28,29]. In many developed countries, rotavirus gastroenteritis exhibits prominent winter seasonality, with infection rates decreasing notably in the summer months [30]. Noroviruses are the primary cause of AGE outbreaks, including those related to foodborne outbreaks. In developed countries, outbreak activity tends to increase during the cooler months of the year [31,32].

The most important and dangerous complications related to AGE are severe dehydration, metabolic acidosis, and electrolyte disturbances [33]. Additionally, AGEs may cause acute kidney injury in children [34], leading to longer hospital stays and increased short-term mortality [35], as well as a higher risk of chronic kidney disease, hypertension, and proteinuria in the long term [36,37].

To date, Italy lacks an active etiological surveillance system for tracking infectious gastroenteritis, and there is limited research on estimating the burden of AGEs and the clinical characteristics of hospitalized patients [38,39].

In the Puglia region, a “Regional System of Surveillance of Hemorrhagic Gastroenteritis in Pediatric Age” has been established [40]. This system is built on a network of pediatricians, including those in outpatient consultancy service, emergency rooms, and hospitals, who report infections such as STEC (Shiga Toxin-producing *Escherichia coli*) and VTEC (Verocytotoxin-producing *Escherichia coli*) as well as hemorrhagic gastroenteritis (GE) from *Campylobacter* spp., *Salmonella* spp., *Shigella* spp./*E. enteroinvasive coli* (EIEC), *Yersinia enterocolitica*, and toxigenic *Clostridium difficile* to provide an epidemiological overview of the circulation of pediatric enteropathogens.

Notably, AGEs from viral and parasitic infections are not reported. However, the specific causative agents of AGE in our local area are not well understood.

This study aims to enhance our understanding of the causes and seasonal patterns of AGE, as well as the blood-related data of hospitalized patients.

## 2. Materials and Methods

A retrospective study was conducted on all patients who were admitted with a diagnosis of AGE (ICD 10 code—9.0—Infectious colitis, enteritis, and gastroenteritis) to the Pediatrics and Infectious Diseases Units of the Vito Fazzi Hospital in Lecce, Italy, between 1 January 2021, and 31 December 2023.

Anonymous data regarding demographic characteristics (age and sex), year and month of admission, length of hospital stay, isolated pathogens, and presence of co-infections were collected from the hospital database.

In addition, blood chemistry data including complete blood count, sodium, potassium, creatinine, ALT, AST, γ-GT, and total bilirubin at the time of admission were collected from all patients.

Stool samples were analyzed using multiplex PRC xTAG Gastrointestinal Pathogen Panel (GPP), Diasorin for the simultaneous qualitative detection and identification of multiple bacteria such as *Campylobacter*, *Clostridium difficile*, *Escherichia coli*, *Salmonella*, *Shigella*, *Vibrio chlolerae* and *Yersinia enterocolitica* as well as Adenovirus, Norovirus, and Rotavirus, and parasitic infections from *Cryptosporidium*, *Entamoeba histolytica*, and Giardia in individuals displaying signs and symptoms of infectious colitis or gastroenteritis. The test exhibited an overall sensitivity of 94.3%.

The xTAG technology is based on nucleic acid amplification by PCR, and the amplified DNA is combined with short-sequence TAG primers. If the target is present, target-specific primer extension occurs, and a marker is incorporated at the same time. The addition of beads to the anti-TAG sequence helps to identify the target primer through complementary pairing.

The study was conducted in compliance with the Declaration of Helsinki and was approved by the Research Ethics Committee (Prot. N.322 of 09/05/2024—Prot.1655/CEL—FoodbornePathogens_LE). Due to the retrospective nature of the study, informed consent was not necessary.

Continuous variables were presented as mean ± standard deviation or median and interquartile range, as appropriate. Categorical variables were described using frequency and percentages. Levene’s test was employed to assess the homogeneity of the data. The Mann–Whitney test was used for non-normally distributed data.

Proportions in categorical variables were compared using the chi-square test, while Fisher’s exact test was used in cases where data were limited.

A *p*-value < 0.05 was considered statistically significant. The statistical analysis was performed using the SPSS (Statistic Package for Social Science) software version 24.0 (IBM Corp., Armonk, NY, USA).

## 3. Results

In the period between 1 January 2021 and 31 December 2023, a total of 103 patients were admitted with the diagnosis of AGE to the Pediatrics and Infectious Diseases Departments of Lecce Hospital.

The patients, with an age range of 0 to 15 years and a median age of 3 years (IQR 1–9) comprised 60 males. Most admissions (61 out of 103 patients) occurred in 2022, with the highest number of patients admitted in autumn (39 patients, 37.86% of the total) and summer (37 patients, 35.92% of the total) (Table 1).

Overall, 78 bacterial, 35 viral, and 7 parasitic infections were detected.

**Figure 1:**
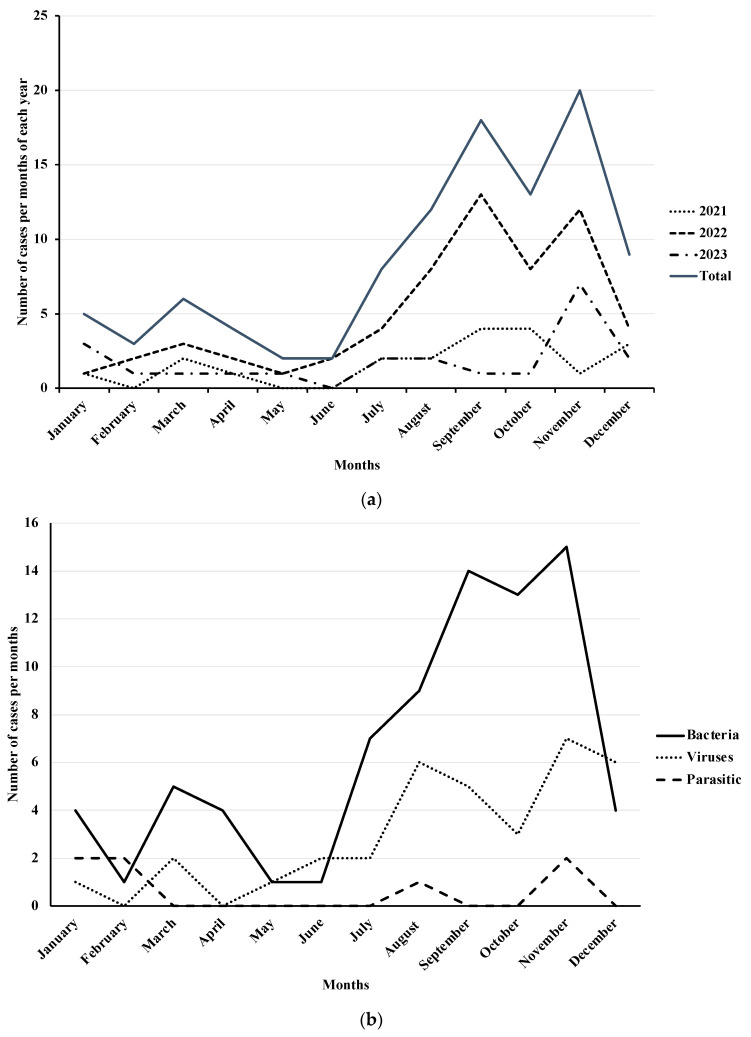
Seasonality of AGE stratified by annual hospitalized cases (**a**) and causative pathogen (**b**) from 2021 to 2023.

The majority of cases occurred in September and November, except in 2021 when the peaks were observed in October and December. Specifically, bacterial infections were most prevalent in September and November, viral ones in August and November, and parasitic infections in September and November (Figure 1).

The patients were divided into two age groups: <5 years (*n* = 59) and 5–15 years (*n* = 44).

All patients were discharged, and no complications, such as alcoholosis, severe dehydration, or sepsis, developed during the disease. The median hospital length of stay was 5 days for both groups, with an interquartile range of 4–6 for the group <5 years and 4–7 for patients aged 5–15 years (Table 2).

Specifically, of the *Escherichia coli* infections, 27 cases were due to Enteropathogenic *Escherichia coli* (EPEC), 9 cases were Enteroaggregative *Escherichia coli* (EAEC), and 4 were Shiga toxin-producing *Escherichia coli* (STEC).

In total, 17 co-infections (16.50%) were identified, of which 10 (16.95%) were in Group 1 and 7 (15.91%) in Group 2.

The comparison between the two groups of patients under and over 5 years of age revealed that bacterial infections are more common among older children, while viral infections are mainly observed in children under 5 years of age. *Campylobacter jejuni* infections were significantly higher in Group 2 while Norovirus infections were higher in Group 1.

However, no significant difference was found for parasitic infections between the two groups (Table 2).

As far as blood chemistry tests are concerned reduced levels of hematocrit at admission were found in 38.32% of total cases, increased levels of lymphocytes in 42.05%, neutrophils in 67.29%, and CRP in 63.55%, without any significant difference between the two groups.

Significantly higher creatinine values (*p* < 0.0000) were found among children under 5 years of age in a percentage of 33.90% compared to 8.33% in patients ≥ 5 years. A significant increase in lymphocytes (*p* < 0.0155) was observed in patients ≥ 5 years with a percentage of 61.36% vs. 37.29% in children less than 5 years of age (Table 3).

**Figure 2:**
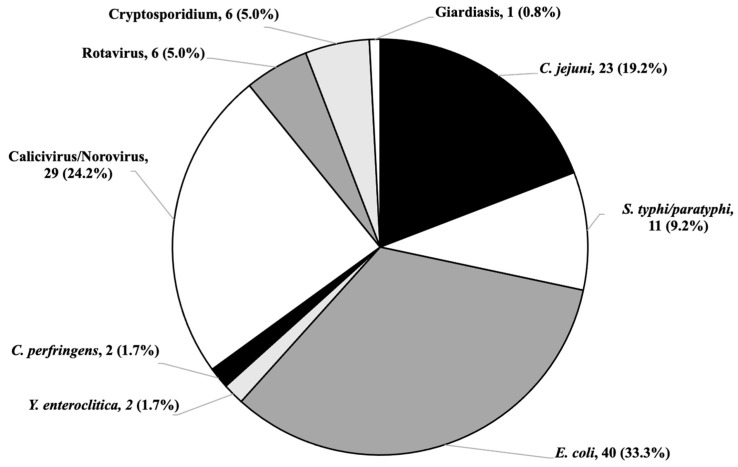
Number and percentage of detected pathogens in the patients with AGE.

The most commonly detected pathogens among bacteria were *Escherichia coli* (40 patients, 33.3% of the total), *Campylobacter jejuni* (23 patients, 19.2% of the total), and *Salmonella typhi/paratyphi* (11 patients, 9.2% of the total), while among viral infections, Norovirus is the most represented (29 patients, 24.2% of the total). Only a few cases of *Cryptosporidium* parasitic infections (6 patients, 5.0% of the total) were detected (Figure 2).

Among the 17 recorded co-infections, the bacterial–viral one characterized by *Escherichia coli* and Norovirus was the most frequently found, especially in younger patients. The bacterial coinfection of *Escherichia coli* and *Campylobacter jejuni* and the bacterial and parasitic coinfections of *Salmonella typhi/paratyphi* + *Cryptosporidium* were only found in patients ≥ 5 years (Table 4).

## 4. Discussion

In high-income countries like Italy, despite the socioeconomic status and high level of sanitation, AGE is still widespread and, although rarely fatal, causes a significant number of visits to the emergency department and hospital admissions [41].

On average, in Italy annually there are over 20,000 hospitalizations for AGE in children under 6 years [42]. The GBD Study in 2021 estimated 3.4 deaths [95% UI = 2.71–3.86] for 100,000 people in Italy due to diarrheal diseases, with 4.28 deaths [95% UI = 3.21–4.99] among females and 2.48 deaths [95% UI = 2.18–2.7] among males [43].

In the Puglia region, from 06/21/2018 to 11/30/2021, there was an occurrence of 30.8 cases of GE per 10,000 children. Specifically, in the Province of Lecce, 160 cases were reported, with an incidence of 14.0 cases/10,000 children [44]. In our study, we found incidences of 17.77, 67.84, and 32.99 cases per 10,000 children in the years 2021, 2022, and 2023, respectively.

These rates are comparable to the regional data, considering that viral and parasitic infections are not included in the regional report.

The patients included in our analysis were mainly admitted to the hospital in 2022 with 61 out of 107 patients being admitted during that year, and with peaks in autumn and summer. The study was conducted between 1 January 2021, and 31 December 2023, during (2021) and immediately after (2022–2023) the COVID-19 pandemic, and the results obtained were somewhat conditioned by the peculiar global situation [45].

The reduced number of hospitalizations for AGE in 2021 may have been influenced by the COVID-19 pandemic, which had a significant impact on hospital admissions, in the southern regions of Italy as well [46,47], as well as by the lockdown and the use of masks, which contributed to reducing the circulation of infectious agents.

In addition to the lockdown, several cities experienced prolonged school closures, including nursery schools, during the pandemic. This situation may have further limited the circulation of pathogens and led to a decrease in the risk of contagion, predominantly among younger children, as evidenced by our data. The return to normal life with the circulation of all infectious pathogens led to a rebound in hospital admissions which then returned to lower levels in the following year.

Our results reveal a seasonal pattern, with viral infections prevalent in autumn and bacterial infections in summer. Other studies have highlighted a prevalence of viral infections during the cold season, with a peak of adenovirus infections in November and astrovirus in December [48].

Viral infections are usually contracted in the community through oral–fecal transmission, with daycares being higher-risk settings, while bacterial infections are typically acquired from undercooked or contaminated food [33,49]. Although recent studies describe the participation of norovirus in cases of acute diarrhea due to contamination of food or water [50,51].

Our findings indicate that bacterial infections are common in both age groups, with *E. coli* and *C. jejuni* infections occurring more frequently. A comparison between the two groups reveals that viral infections are significantly more prevalent among children under 5 years old, while bacterial infections are more common in older individuals. Viral infections are frequently acquired during the school period, suggesting that they could be caused by infections contracted within the community.

Bacterial infections are primarily observed in older children, likely as a result of consuming contaminated or undercooked foods.

Based on the date of hospitalization and the identified causative agents, we found no cases that could be attributed to a local outbreak.

Our data are in line with other studies confirming that viral gastroenteritis is prevalent in children [38,52,53], and it accounts for at least 50% of all cases of AGE [54].

Similar results were reported in a recent German review, which indicated that acute infectious gastroenteritis remains the second most common non-traumatic cause of emergency hospital admission in children aged 1 to 5 years. This is primarily caused by viruses with 47% being rotavirus, 29% norovirus, and 14% adenovirus [55].

Furthermore, the hospital length of stay (median length of 5 days without any difference between the ages) seems consistent with findings from other studies, even outside the national territory [52]. Comparing the two subgroups of patients, it was found that a significantly higher number of patients of Group 1 had elevated creatinine values compared to these in Group 2. Creatinine is a byproduct of muscle protein metabolism and elevated levels may indicate infection, acute kidney injury [56], and dehydration severity [57].

Young children are more vulnerable to dehydration than older children or adults due to their higher metabolic rates, inability to communicate their needs or hydrate themselves, and increased insensible losses [58].

The main etiological agents found in all hospitalized patients are *Escherichia coli* (39.3%*)*, Norovirus (27.1%), and *Campylobacter jejuni* (21.5%) with greater prevalence of *Escherichia coli* and *Campylobacter jejuni* among patients over 5 years of age and *Escherichia coli* and Norovirus among the younger group. Few cases of hospitalizations for *Salmonella typhi*/*paratyphi* (12.5%) and rotavirus (5.6%) were found in both groups.

All detected pathogens can be transmitted in similar ways, including through animal–human interaction, consumption of contaminated food or drink, contact with food handlers who are ill, and transfer via contaminated surfaces and utensils. To reduce the risk of these diseases, appropriate preventive strategies are needed, which include adequate sanitation, food hygiene and targeted health education, as well as the greater use of existing vaccines and the development of new ones against circulating microorganisms.

Before the introduction of the vaccine, rotavirus was the main etiological agent of gastroenteritis, mainly in children. As shown in an epidemiological study conducted in Salento, rotavirus infection represented an important cause of hospitalization [59]. Vaccination has resulted in an important reduction in mortality, hospitalization rates, and overall number of infections [60,61]. The global impact of immuno-prophylaxis is evident, with a reduction of 40% in infection incidence, as shown by a study performed in several countries participating in the Global Rotavirus Surveillance Network [62]. Italy has approved two oral live attenuated rotavirus vaccines: RotaTeq^®^ (MSD Canada Inc., Rahway, NJ, USA) and Rotarix^®^ (GlaxoSmithKline, Brentford, UK). RotaTeq^®^ is a 5-valent human–bovine reassortant vaccine. It should be administered between 6 and 12 weeks of age and requires three doses with a recommended interval of one month between each dose. The Rotarix^®^ is a monovalent vaccine that requires two doses, with at least one month between each other. It should be given between 6 and 15 weeks of age.

In Apulia, vaccination has been available since 2015 upon direct request from parents. In 2022, Apulia reported a rotavirus vaccination coverage of 79.45% for newborns, exceeding the national coverage of 74.39% [63]. Real-world evidence has shown sustained protection against rotavirus disease, especially against severe gastroenteritis [64].

Regarding the development of new vaccines against the microorganisms detected in our analysis, many vaccines against ETEC have been tested for safety and immunogenicity. Only one recombinant intradermal vaccine has completed phase 2 clinical trials and another inactivated oral administration has completed phase 3.

Moreover, developing effective and safe vaccine candidates against noroviruses is challenging due to their high genetic variability, which includes 5 genogroups comprising 35 genotypes. Noroviruses cannot be cultured on cells, so a cell culture-based neutralization assay is not possible. Additionally, there are no known correlates of protection against infection and disease. As a result, vaccines using virus-like particles (VLPs), recombinant technologies, and viral vectors are currently under development and testing. As of now, only two vaccine candidates are in phase 3 testing. It is necessary to await the results of these studies to determine whether they will be the first effective and safe commercial vaccines against noroviruses.

In Italy, there is no specific nationwide surveillance system for all cases of AGE, except for Salmonellosis and STEC. As a result, the etiological agents of AGE among patients primarily rely on individual healthcare institutions to be conducting research. Our study is one of the first in our region to investigate the spread of the main pathogens responsible for AGE-related hospitalizations.

While most patients suffering from gastroenteritis are treated as outpatients, our study focused on hospitalized subjects. Therefore, it provides limited knowledge about the overall incidence of gastroenteritis and its causes in the general population.

Our patients were tested for most of the pathogens responsible for AGE, but not for all, such as Parechovirus, Aichivirus, Bocavirus, and Sapovirus, which also play an important pathogenic role in AGE [8,65].

Furthermore, the study is based on data from a single hospital and lacks a lot of clinical information about patients, such as symptoms and signs, vaccination status, and drug therapy.

## 5. Conclusions

Our data describe the etiological agents that caused hospitalization for AGE in two age groups and can serve as a basis for further investigations of the microorganisms responsible for AGE-related hospitalizations, including information about the symptoms and clinical aspects, that were not covered in this study. Our findings may provide evidence to support interventions aimed at preventing foodborne illness.

AGE is a serious health concern that could be effectively prevented by implementing food-based and community-level sanitation systems, as well as strengthening vaccination policies and developing new effective and safe vaccines to reduce the spread of these diseases with significant cost savings.

## Figures and Tables

**Table 1:** Characteristics of hospitalized patients (*n* = 103) with AGE.

Characteristics	All Patients (*n* = 103)	Group 1 (<5 Years) (*n* = 59)	Group 2 (≥5 Years) (*n* = 44)	*p*
Age, median years (IQR)	3 (1–8)	1 (1–2)	9 (6–12.25)	0.0001 ^
Male, *n*. (%)	60 (58.25)	33 (55.9)	27 (61.36)	0.5803 **
Hospital admission year, *n*. (%)				
- 2021, *n*. (%)	20 (18.69)	12 (20.34)	8 (18.18)	
- 2022, *n*. (%)	61 (57.01)	39 (66.10)	22 (40.00)	
- 2023, *n*. (%)	26 (24.30)	8 (13.56)	14 (31.82)	0.0782 **
Hospital admission season (2021–2023), *n*. (%)				
- Summer, *n*. (%)	37 (35.92)	20 (33.90)	17 (38.64)	
- Autumn, *n*. (%)	39 (37.86)	26 (44.07)	13 (29.55)	
- Winter, *n*. (%)	19 (18.45)	10 (16.95)	9 (20.45)	
- Spring, *n*. (%)	8 (7.77)	3 (5.08)	5 (11.36)	0.3903 **
Hospital length of stay in days, median (IQR)	5 (4–6)	5 (4–6)	5 (4–7)	0.2762 ^

** Chi-square test; ^ Mann–Whitney test (independent sample).

**Table 2:** Diagnosis of hospitalized patients for AGE.

Diagnosis	Group 1 (<5 Years) (*n* = 59)	Group 2 (≥5 Years) (*n* = 44)	*p*
**Method**			
Coproculture, *n*. (%)	4 (6.78)	1 (4.54)	
Multiplex PCR, *n*. (%)	55 (93.22)	43 (97.72)	0.3898 *
**Microbiological pathogen detected**			
**Bacterial infection**, *n*. (%)	40 (67.80)	42 (95.45)	0.0004 *
*C. jejuni*, *n*. (%)	8 (13.56)	15 (34.09)	0.1332 **
*S. typhi*/*Paratyphi*, *n*. (%)	5 (8.47)	6 (13.64)	0.4014 **
*E. coli*, *n*. (%)	25 (42.37)	15 (34.09)	0.3936 **
*Y. enterocolitica*, *n*. (%)	1 (1.69)	1 (2.27)	1.0000 *
*C. perfringens*, *n*. (%)	1 (1.69)	1 (2.27)	1.0000 *
**Viral infection**, *n*. (%)	26 (44.07)	9 (20.45)	0.0123 **
Norovirus, *n*. (%)	23 (38.98)	6 (13.63)	0.0047 **
Rotavirus, *n*. (%)	3 (5.08)	3 (6.81)	0.6989 *
**Parasitic infection**, *n*. (%)	3 (5.08)	5 (11.36)	0.2820 *
Cryptosporidium, *n*. (%)	3 (5.08)	4 (9.09)	0.4567 *
Giardiasis, *n*. (%)	0 (-)	1 (2.27)	0.4272 *
**Co-infections, *n*. (%)**	10 (16.95)	7 (15.91)	0.8881 **

* Fisher’s exact test; ** Chi-square test.

**Table 3:** Hematology and serum chemistry value of the hospitalized patients for AGE.

Hematology and Serum Chemistry Value	Group 1 (<5 Years) (*n* = 59)	Group 2 (≥5 Years) (*n* = 44)	*p*
Decreased Hematocrit	22 (37.29)	17 (38.63)	0.8890 **
Increased Lymphocytes	22 (37.29)	27 (61.36)	0.0155 **
Increased Neutrophils	40 (67.80)	29 (65.90)	0.9918 **
Increased CRP	36 (61.02)	29 (65.90)	0.3605 **
Decreased Sodium	23 (38.98)	22 (50.00)	0.3605 **
Decreased Potassium	9 (15.25)	3 (6.82)	0.2274 *
Increased Creatinine	20 (33.90)	4 (8.33)	0.0000 *
Increased AST	3 (5.08)	0 (-)	0.2587 *
Increased ALT	1 (1.69)	0 (-)	1.0000 *
Increased γ-GT	0 (-)	0 (-)	1.0000 *
Increased Total bilirubin	0 (-)	0 (-)	1.0000 *

Hematocrit; normal value: 38–48%. Lymphocytes; normal value: 1000–3000 × 10^3^/μL. Neutrophils; normal value: 1500–7000 × 10^3^/μL. CRP, C-reactive protein; normal value: <10 mg/dL. Sodium; normal value: 135–150 mmol/L. Potassium; normal value: 3.5–5.5 mmol/L. Creatinine; normal value: 0.67–1.20 mg/dL. AST, aspartate aminotransferase; normal value: <45 UI/L. ALT, alanine aminotransferase; normal value: <45 UI/L. γ-GT, gamma-glutamyl transferase; normal value: 5–50 UI/L. Total bilirubin; normal value: <1.25 mg/dL. * Fisher’s exact test; ** Chi-square test.

**Table 4:** Mixed infections identified in patients with acute gastroenteritis.

Mixed Pathogens	All Patients (*n* = 103)	Group 1 (<5 Years) (*n* = 59)	Group 2 (≥5 Years) (*n* = 44)
**Bacterial**			
*E. coli* + *S. typhi*/*Paratyphi*, *n*. (%)	2 (1.94)	1 (1.69)	1 (2.27)
*E. coli* + *C. jejuni*, *n*. (%)	3 (2.91)	(-)	3 (6.81)
**Bacterial + viral**			
*E. coli* + Norovirus, *n*. (%)	6 (5.82)	5 (0.72)	1 (2.27)
*C. jejuni* + Rotavirus, *n*. (%)	1 (0.97)	1 (1.69)	(-)
*C. jejuni* + Norovirus, *n*. (%)	1 (0.97)	(-)	1 (2.27)
*Y. enterocolitica* + Rotavirus, *n*. (%)	1 (0.97)	1 (1.69)	(-)
**Bacterial + parasitic**			
*E. coli* + *Cryptosporidium*, *n*. (%)	2 (1.94)	2 (3.39)	(-)
*S. typhi*/*Paratyphi* + *Cryptosporidium*, *n*. (%)	1 (0.97)	(-)	1 (2.27)

## Data Availability

Anonymized data will be made available on request by any qualified investigator under the terms of the registries’ usage and access guidelines and subject to the informed consent of the patients.
